# A Barcode-Based Phylogenetic Characterization of *Phytophthora cactorum* Identifies Two Cosmopolitan Lineages with Distinct Host Affinities and the First Report of *Phytophthora pseudotsugae* in California

**DOI:** 10.3390/jof8030303

**Published:** 2022-03-16

**Authors:** Tyler B. Bourret, Sebastian N. Fajardo, Cole P. Engert, David M. Rizzo

**Affiliations:** Department of Plant Pathology, University of California, Davis, Davis, CA 95616, USA; snfajardo@ucdavis.edu (S.N.F.); cengert@ucdavis.edu (C.P.E.); dmrizzo@ucdavis.edu (D.M.R.)

**Keywords:** *Phytophthora cactorum*, *Phytophthora pseudotsugae*, *Phytophthora hedraiandra*, *Phytophthora* ×*serendipita*, *Fragaria*, *Malus*, *Quercus*, California, phylogeny, hybrid

## Abstract

A collection of 30 *Phytophthora cactorum* and 12 *P. pseudotsugae* (subclade 1a) strains isolated from several recent surveys across California was phylogenetically compared to a worldwide collection of 112 conspecific strains using sequences from three barcoding loci. The surveys baited *P. cactorum* from soil and water across a wide variety of forested ecosystems with a geographic range of more than 1000 km. Two cosmopolitan lineages were identified within the widespread *P. cactorum*, one being mainly associated with strawberry production and the other more closely associated with apple orchards, oaks and ornamental trees. Two other well-sampled *P. cactorum* lineages, including one that dominated Californian restoration outplantings, were only found in the western United States, while a third was only found in Japan. Coastal California forest isolates of both *Phytophthora* species exhibited considerable diversity, suggesting both may be indigenous to the state. Many isolates with sequence accessions deposited as *P. cactorum* were determined to be *P. hedraiandra* and *P. ×serendipita*, with one hybrid lineage appearing relatively common across Europe and Asia. This study contains the first report of *P. pseudotsugae* from the state of California and one of the only reports of that species since its original description.

## 1. Introduction

*Phytophthora* is a genus of oomycete plant pathogens with the potential to cause great harm to plants in both agriculture and forests. *Phytophthora cactorum* (Leb. and Cohn) Schroeter (1886) is one of the oldest and best-known *Phytophthora* species, having originally been described as *Peronospora cactorum* in 1870 before the establishment of *Phytophthora* in 1876 [1,2]. *Phytophthora cactorum* has long been recognized as a cosmopolitan species with a wide host range, particularly associated with orchard crown and fruit rots, and remains an important agricultural pest [3,4,5].

In a genus with few reliable morphological characteristics for species diagnostics, *P. cactorum* remained literally in its own morphological “group” for most of the 20th century due to the species’ unique combination of homothallism with largely paragynous antheridia and papillate, deciduous, externally proliferating sporangia [6]. In 1983, *Phytophthora pseudotsugae* (Hamm and Hansen) was separated from *P. cactorum* and described based on subtle but reliably distinct morphological characteristics. In contrast to *P. cactorum*, *P. pseudotsugae* appears to have a narrow host range, having only been found to cause a root rot of *Pseudotsugae menziesii* and *Abies* spp. in Washington and Oregon, USA [2,7,8]. In 1995, *P. idaei* was described [9,10] and, along with *P. cactorum* and *P. pseudotsugae*, the three similar-looking species were soon identified as forming a phylogenetic clade [11]. This clade was designated subclade 1a [12]. Since 2000, *P. hedraiandra*, *P. aleatoria* and *P. alpina* have been added to subclade 1a, as well as the hybrids *P. ×serendipita* (*P. cactorum × hedraiandra*) and *P. ×pelgrandis* (*P. cactorum × nicotianae*) [13,14,15,16,17].

Much research has been conducted regarding the host preferences of individual isolates of *P. cactorum*. Before molecular genetic characterization was feasible, researchers noticed varying levels of aggressiveness when strains of *P. cactorum* isolated from different hosts were tested on a common host [18,19]. Restriction enzyme-based molecular studies identified considerable intraspecific genetic diversity, excepting the majority of isolates from *Fragaria* which appeared to derive from a relatively clonal lineage [10,20,21,22,23]. This early molecular evidence and pathogenicity trials suggested that the *P. cactorum* isolates capable of causing strawberry crown rot are phylogenetically distinct from those causing leather (fruit) rot, and that the former are also more capable pathogens on a variety of other hosts. These findings were recently confirmed by Nellist et al. (2021) [24], finding a strawberry crown rot-associated clade; isolates from outside that clade were less aggressive on strawberry crowns, and isolates from within the strawberry lineage were less aggressive on apple.

Pánek et al. (2016 & 2021) [25,26] found higher genetic diversity in European strains of *P. ×serendipita* than of *P. cactorum*, concluding that *P. cactorum* was not indigenous to Europe; Jung (2009) [27] found *P. cactorum* to have a largely urban distribution within Bavaria. Based on the inclusion of many isolates from New York state, Eikemo et al. (2004) [20] suggested that the leather rot-associated, wider host-range lineage, may have originated in North America; Bhat et al. (2006) [23] saw considerable intraspecific diversity within a collection of mostly agricultural isolates from California. Over the past 20 years of field work in California, we have amassed a large collection of *P. cactorum* and *P. pseudotsugae* strains. These strains have come from a range of native plant communities (Table 1). Our *P. cactorum* strains exhibited intraspecific variability based on the primary barcoding locus, the internal transcribed spacer of the ribosomal DNA repeat (ITS rDNA), and these differences appeared to correlate somewhat with different projects and land-uses across California.

The overall objective of this study was to compare these putative intraspecific *P. cactorum* lineages from California to a worldwide sample of *P. cactorum*. This was done by sequencing two additional loci known to be more rapidly-evolving than ITS, “cox1” (mitochondrially-encoded cytochrome c oxidase subunit I, COI or COX1) [28,29] and “cox2+spacer” (cox2, cytochrome c oxidase subunit II, along with the intergenic spacer between cox1 and cox2) [30,31] from a selection of 30 *P. cactorum* isolates with five different ITS variants. We also included 12 putative *P. pseudotsugae* isolates from California to support their phylogenetic placement and investigate the hypothesis that both *P. cactorum* and *P. pseudotsugae* may be indigenous to western North America. To place our *P. cactorum* isolates within a worldwide species, we obtained cox1 and/or cox2+spacer data in the GenBank nucleotide collection sequenced from 86 *P. cactorum* strains isolated across six continents, augmenting this with genome-sequencing data from an additional 21 strains.

## 2. Materials and Methods

### 2.1. Sources of California Isolates

**Figure 1:**
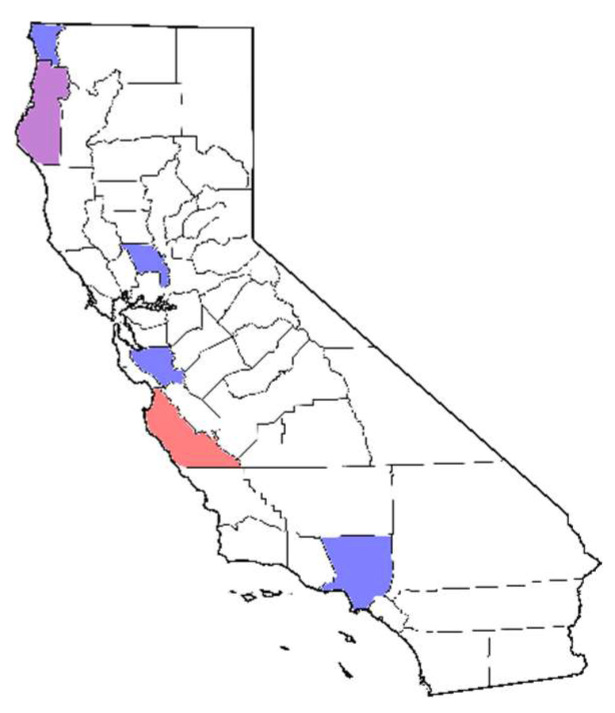
California counties (Co.) where *Phytophthora* strains were collected for this study, shaded blue for *P. cactorum*, red for *P. pseudotsugae* and purple for both species. From north (top) to south (bottom), Del Norte Co. (7 strains), Humboldt Co. (2 strains), Yolo Co. (2 strains), Santa Clara Co. (8 strains), Monterey Co. (11 strains) and Los Angeles Co. (12 strains).

The isolates for this study originate from several projects conducted during the last decade (Table 1) over a geographic range of more than 1000 km within California (Figure 1). Isolations were plated into semi-selective media CMA-PARP (corn meal agar amended with pimaricin, ampicillin, rifampicin and pentachloronitrobenzene, and sometimes hymexazol) [32]. All strains are stored as water vials [33] in our long-term isolate collection at the University of California, Davis (UCD).

**Restoration outplantings.** The largest number of *P. cactorum* isolates were collected from surveys of restoration outplantings in California. A 2015–2016 survey in Santa Clara County in the southern San Francisco Bay-area found *P. cactorum* to be the most common and widespread species [32], and the eight isolates in this study represent only a subsampling of the *P. cactorum* isolates obtained in that survey. *P. cactorum* is also one of the species most commonly encountered in surveys of restoration areas within the Angeles National Forest (ANF) in southern California [34]. Isolates from these surveys were obtained via soil baiting: soil (and sometimes soil and root) samples were collected beneath plants, transported to the laboratory, and baited with pear fruit and rhododendron leaves.

**Stream monitoring.** As part of a *Phytophthora ramorum* stream-monitoring program in coastal California, other species of *Phytophthora* have been isolated and saved [35,36]. This includes 8 strains of *P. cactorum* between 2013 and 2015 and a single isolate of *P. pseudotsugae* from 2010 (Table S1). Isolates were collected by deploying rhododendron leaf bait into coastal streams during the spring and early summer [35,37,38]. Leaves were left in the running water for several weeks and surface-sterilized in 10% bleach prior to isolations.

**Chapparal and stream soil baiting.***Phytophthora* surveys have been conducted in recently-burned ANF chaparral and woodlands, including riparian vegetation not associated with ecosystem restoration [34]. These surveys employed the same sampling methods as the restoration outplantings.

**Forest soil baiting.** As part of a survey for sudden oak death, soils were sampled from a plot network in the Big Sur region of California [39]. In 2013, 40 plots, dominated by coast redwood (*Sequoia sempervirens*), were visited, and 9 soil monoliths ~0.5 L were randomly collected within each 500 m^2^ plot. Soil samples of ~50 g were placed in plastic tubs and baited with rhododendron leaves. Ten isolates of *P. pseudotsugae* were baited with this method, along with 7 isolates of *P. ramorum*, 6 of *P. pseudosyringae*, and one each of *P. chlamydospora*, *P. nemorosa* and *P. syringae* (unpublished data).

**Direct sampling of seedling roots.** In summer 2015, seedling fine roots were sampled from within the same Big Sur plot network, yielding a single *P. pseudotsugae* isolated directly from thoroughly-rinsed roots of *Notholithocarpus densiflorus* (tanoak).

**UCD campus baiting.** Two *P. cactorum* isolates were collected on UCD campus in California’s central valley, where Mircetich et al. (1977) [40] had previously isolated *P. cactorum* from declining, nursery-origin *Quercus agrifolia* (coast live oak). In 2012 and 2016, soil samples were taken beneath the same declining *Q. agrifolia* specimen and baited with rhododendron leaves (2012) and rhododendron leaves and pear fruit (2016) (Table 1).

### 2.2. DNA Extraction, Amplification & Sequencing

ITS rDNA and mt cox2-cox1 sequences were obtained as described by Bourret et al. (2018) [41]. Briefly, isolates were grown in 1 mL of pea broth, then DNA was extracted using PrepMan Ultra according to manufacturer’s instructions. ITS sequences were amplified and sequenced using the primers FRiz+ITS4TT [41], while the mitochondrial contig between primers FM75 and FM83 [29] (i.e., cox2-cox1) was obtained using two overlapping sets of primers: FM75+COXFRizA for the cox2+spacer and COXFRizB+FM83 for the cox1. For the cox2+spacer, primer FM78 was employed as an internal reverse primer to ensure complete coverage of the partial cox2 CDS, and COXFRizC as an internal forward primer if homopolymers in the spacer region caused downstream issues with COXFRizA runs [41]. Mitochondrial-encoded ribosomal protein S10 (rps10) sequences were obtained using primers rps10-F and rps10-R [42]. Sanger sequencing was performed by the UC Davis DNASeq facility, and the data assembled using the Chromaseq plugin of Mesquite [43,44]. Sequences were deposited into the NCBI nucleotide collection (Table S1).

### 2.3. Data Assembly & Phylogenetic Inference

Comparable *P. cactorum* and *P. pseudotsugae* isolates with sequence accessions in the nucleotide collection were discovered using separate BLAST searches of the cox2+spacer and cox1 loci as well as text searches of the NCBI Nucleotide collection. The NCBI Genomes page was searched for *P. pseudotsugae* and *P. cactorum*, revealing 21 *P. cactorum* strains with genome-sequencing data available in GenBank (Table S1). Sequences were obtained from the sequence read archive data as described by [41], and in the case of one strain, from the assembly data (Table S1). The cox2+spacer sequence of the ex-type strain of *P. aleatoria* was also obtained from genome-sequencing data.

Sequences from all three loci (i.e., ITS rDNA, mitochondrial cox2+spacer and mt cox1) were available for 72 subclade 1a strains: 42 California isolates from this study, 21 *P. cactorum* strains with publicly-available genomes, four *P. cactorum* and two *P. pseudotsugae* strains from GenBank nucleotide collection with complete coverage (including ex-type strains of both species), as well as the ex-type strains of *P. aleatoria*, *P. hedraiandra* and *P. idaei* (Table S1). *Phytophthora iranica* (subclade 1b) and *P. ipomoeae* (subclade 1c) were used as outgroups to produce a complete-coverage, 74-isolate alignment.

A partial-coverage, multi-locus data set of 169 isolates including outgroups was also constructed. Added to the 74-isolate alignments were an additional 76 *P. cactorum* strains, three *P. pseudotsugae* strains and the ex-type strains of *P. alpina* and *P. ×serendipita*, which had cox1 sequences available in the nucleotide collection but no cox2+spacer sequence, although six did have cox2-only sequences available (Table S1). Six additional *P. cactorum* strains had cox2+spacer sequences in the collection without any accompanying cox1, and eight strains had only the cox2-cox1 spacer available. Of the 95 additional isolates with mitochondrial sequence data, all but 11 also had accompanying ITS sequences. In this larger data set there was just over 50% coverage of the cox2+spacer locus, and nearly full coverage of ITS and cox1. The loci were also analyzed individually (Figures S1–S3).

Data sets were assembled and aligned within AliView [45] using MAFFT [46,47,48], as described by [41]. Phylogenetically informative insertion/deletion data were encoded as separate alignments of binary characters for the cox2+spacer and ITS alignments using FastGap [49]. Maximum likelihood phylogenetic inference was conducted with IQTREE 2.1.2 [50,51,52] using the following options: --merit -AICc (use corrected Akaike information criterion for model-testing) -merge greedy -merge-model all -merge-rate all -Q (use the greedy algorithm of PartitionFinder2 [53] to compare partition schemata and test all possible evolutionary models, with unlinked branches) -allnni (use a more thorough best-tree search) -polytomy (collapse zero-length branches) -b 1000 (perform 1000 non-parametric bootstrap replicates). Loci were partitioned according to sequence features, with ITS1, 5.8S rRNA and ITS2 partitioned for ITS, the cox2+spacer partitioned into cox2 CDS (by codon position), small spacer, “orf32” CDS (by codon position) and the large spacer, and the cox1 CDS by codon position. This resulted in a total of 16 partitioned subsets including two binary gap subsets, which (when option -Q was invoked to allow unlinked branch lengths across partitions) were combined into a single nucleotide subset (model: TVM+F+R2) and a single binary subset (model: JC2+FQ+ASC) by the PartitionFinder2 function of IQTREE for the 169-isolate data set. In the 74-isolate data set, there were two nucleotide subsets, one containing the cox2 CDS 3rd codon position, the “orf32” CDS 3rd codon position, cox1 CDS 3rd codon position, and the small cox2 spacer (model: TIM3+F+R2) and a second containing the rest of the nucleotide subsets (model: GTR+F+I). Bayesian trees were also obtained for the two multi-locus data sets, omitting the gap partitions, and using PartitionFinder2 with linked branch lengths, as described by [41]. Trees were visualized and support values from Bayesian and ML analyses combined using TreeGraph2 [54], followed by annotations with InkScape (inkscape.org). The multi-locus alignments, along with ITS and cox2-cox1 sequences derived from genome sequencing data were deposited in a Dryad repository at doi:10.25338/B8J33M. Split-decomposition (SDN) and median-joining networks (MJN) were constructed from the 74-isolate data set. The methods of [55] were followed employing SplitsTree4 [56,57] for SDNs and Network 10 (fluxus-engineering.com, accessed on 23 January 2022) [58] for MJNs, and analyzing nuclear and mitochondrial loci separately. Outgroups were omitted for the nuclear data set and only *P. cactorum*, *P. hedraiandra* and *P. pseudotsugae* were included in the mitochondrial data set. The 14 bp and 6 bp insertions in the cox2-cox1 spacer of strains LA266_L3 and 268, respectively, were each shortened to a single bp for the MJN analysis.

## 3. Results

### 3.1. Multi-Locus Genotypes

Multi-locus genotypes of the combined ITS, mitochondrial cox2+cox1 and mitochondrial rps10 loci, with a combined length of ~3600 bp were obtained for 42 California isolates (Table S1). Intraspecific sequence variants were observed at each locus, except that identical ITS sequences were retrieved from the 12 *P. pseudotsugae* isolates; the 30 *P. cactorum* isolates yielded five distinct ITS haplotypes. Ten *P. cactorum* cox2-cox1 genotypes were observed, and nine for *P. pseudotsugae*; three rps10 genotypes were observed for each species. In some cases, a mitochondrial genotype was associated with more than a single ITS haplotype. There were several amino acid substitutions observed within the cox2 CDS alignment, including for several California isolates of both species. All California isolates had identical cox1 amino acid sequences (but not identical nucleotide sequences).

The 30 California *P. cactorum* isolates yielded 15 distinct multi-locus genotypes, while nine distinct multi-locus genotypes were derived from the 12 *P. pseudotsugae* isolates. The most common *P. cactorum* haplotype was CAC3-2-F, with nine isolates from restoration outplantings in both northern and southern California and a single isolate from a native plant nursery. The next most common multi-locus genotype, CAC2-4-D, was shared by four of the stream-baited isolates, including three strains isolated at different sampling times from a single site in Del Norte County. Of the 12 *P. cactorum* strains with unique genotypes, four each were from stream-baiting and restoration outplantings. The single stream-baited, north-coast isolate of *P. pseudotsugae* had the same genotype as the central-coast tanoak isolate, PTS1-13-A. The three *P. pseudotsugae* isolates collected from the same 500 m^2^ plot, TB191, TB192 & ABS-BS-2015(140) all exhibited distinct genotypes from each other, as did the two *P. cactorum* isolates collected under the same *Quercus agrifolia* tree on the UCD campus. This is the first published report of *P. pseudotsugae* in California, the first report of baiting the species from a body of water, and the first isolation of *P. pseudotsugae* from an angiosperm host, tanoak.

### 3.2. Full-Coverage, 74-Isolate Multi-Locus Trees

**Figure 2:**
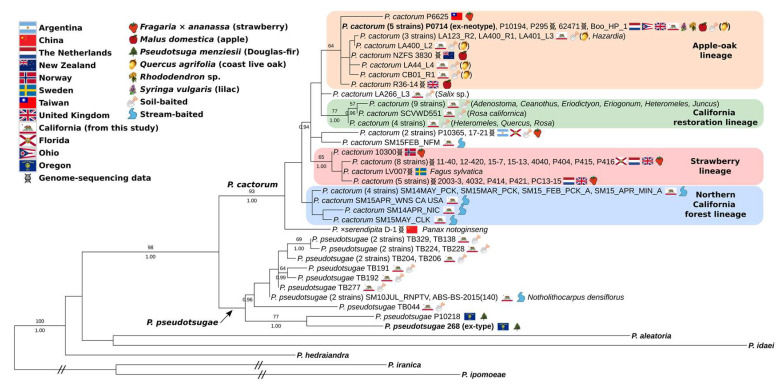
The 74-isolate, multi-locus maximum likelihood tree of *Phytophthora* subclade 1a. Tree inferred with IQTREE2 2.1.2 from ITS rDNA, mt cox2+spacer and mt cox1 loci, with a total of 2932 characters. Support values above branches are percentages ≥50 from 1000 non-parametric bootstrap replicates, and Bayesian posterior probabilities ≥0.90 from a separate analysis in MrBayes 3.2.7. Tree edited in TreeGraph2 and InkScape. Isolates on the same node were collapsed; slashes indicate branches that were artificially shortened for display purposes. Sequence accessions are listed in Table S1. When the shovel icon is followed by a host in parentheses, it indicates an isolate soil-baited from beneath that host.

The results of the 74-isolate multi-locus inference found several distinct intraspecific lineages within *P. cactorum*, with clear distinctions between geographic ranges and isolation sources (Figure 2). Three lineages corresponded to clades with significant support values. Two intraspecific lineages had cosmopolitan distributions (Figure 2). One intraspecific lineage was composed mostly of strawberry (*Fragaria × ananassa*) isolates with no isolates from California. The other widely distributed intraspecific lineage comprises many apple (*Malus domestica*), California oak (*Quercus* spp.) and other isolates. This apple–oak lineage also contains the ex-neotype of *P. cactorum*. Two other intraspecific lineages were composed entirely of California isolates but with different land-uses. One lineage consisted of stream-baited isolates from the forested, coastal, northernmost reaches of the state (northern California forest lineage) and the other from restoration outplantings in both northern and southern California (California restoration lineage). Southern California restoration outplantings also had isolates from the apple–oak lineage, while the outplantings in the Bay area had only isolates from the California restoration lineage.

The clades corresponding to *P. cactorum* + *P. pseudotsugae* and *P. cactorum* had strong support, but not the clade corresponding to *P. pseudotsugae* (Figure 2). Within the *P. pseudotsugae* clade, Oregon and California isolates were reciprocally monophyletic with significant support. Based on its placement and the comparison of the single-locus trees, the Chinese strain D-1, from *Panax notoginseng*, was determined to be *P. ×serendipita* and not *P. cactorum*, as originally designated in GenBank (Figure 2). Our results do not support the hypothesis that *P. hedraiandra* and *P. cactorum* may be conspecific [25,26] unless the entire subclade is considered a single species.

### 3.3. Partial-Coverage, 169-Isolate Multi-Locus Trees

**Figure 3:**
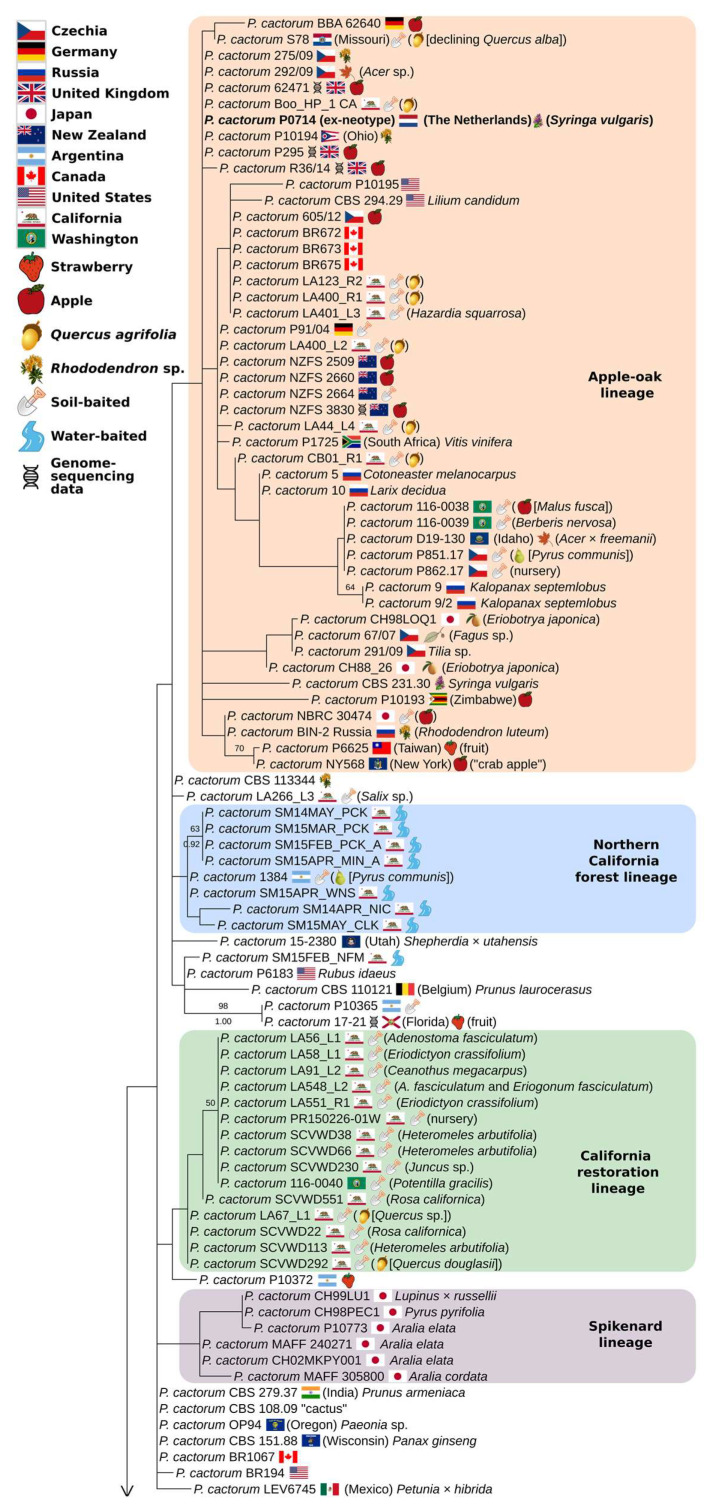
The 169-isolate, multi-locus maximum likelihood tree of *Phytophthora* subclade 1a (top). Tree inferred with IQTREE2 2.1.2 from ITS rDNA, mt cox2+spacer and mt cox1 loci, with a total of 2936 characters. Near-zero branches were collapsed with gotree (github.com/evolbioinfo/gotree, accessed on 31 December 2021). Support values above branches are percentages ≥50 from 1000 non-parametric bootstrap replicates, and Bayesian posterior probabilities ≥0.90 from a separate analysis in MrBayes 3.2.7. Tree edited in TreeGraph2 and InkScape. Slashes indicate branches that were artificially shortened for display purposes. Sequence accessions are listed in Table S1.

**Figure 4:**
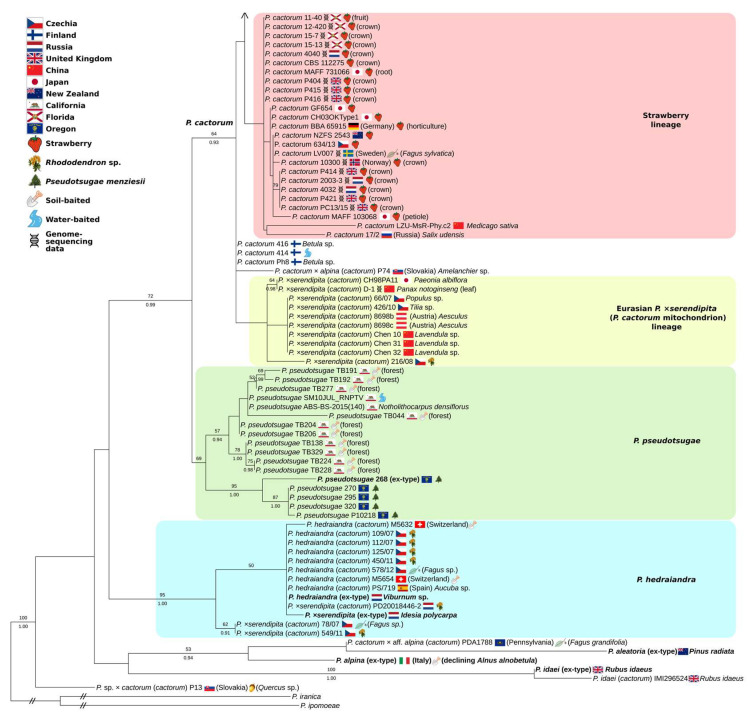
The 169-isolate, multi-locus maximum likelihood tree of *Phytophthora* subclade 1a (bottom). Tree inferred with IQTREE2 2.1.2 from ITS rDNA, mt cox2+spacer and mt cox1 loci, with a total of 2936 characters. Near-zero branches were collapsed with gotree (github.com/evolbioinfo/gotree, accessed on 31 December 2021). Support values above branches are percentages ≥50 from 1000 non-parametric bootstrap replicates, and Bayesian posterior probabilities ≥0.90 from a separate analysis in MrBayes 3.2.7. Tree edited in TreeGraph2 and InkScape. Slashes indicate branches that were artificially shortened for display purposes. Sequence accessions are listed in Table S1.

The 169-isolate, partial-coverage multi-locus tree was congruent with the 74-isolate tree, albeit with lower support values, and augmented the membership of the four previously-identified subspecific lineages (Figure 3 and Figure 4). Additional isolates placed in the greatly expanded apple–oak lineage gave it a much wider geographic range, including South Africa, Zimbabwe, and New Zealand. One additional *Quercus* isolate, several more *Rhododendron* and many additional isolates from *Malus* were added to the expanded lineage, but isolates from *Acer*, *Kalopanax*, *Larix*, *Lilium*, *Syringa*, *Tilia*, and *Vitis* demonstrate the wide host range of this lineage and suggest ornamental plant production, alongside apple production, as a possible worldwide vector; four of the six California *Quercus agrifolia* isolates in this lineage were baited from nursery-origin oaks, while the other two were from natural recruits growing in southern California. Another widespread, early diverging *P. cactorum* lineage was associated with *P. ×serendipita* strains possessing *P. cactorum*-like mitochondrial sequences and *P. hedraiandra*-like ITS sequences. On the cox1-only tree, these strains corresponded to three different cox1 genotypes, with the most common genotype corresponding to the strawberry lineage, and the other genotypes corresponding to a ginseng isolate D-1 from China and rhododendron isolate 216/08 from Czechia. Two strains could not be determined to a described species: P13, from *Quercus*, Slovakia, appears to be a hybrid between an undescribed species and *P. cactorum*, while PDA1788, from *Fagus*, Pennsylvania [59], appears to be a hybrid between *P. cactorum* and an undescribed relative of *P. alpina* (Figure 4 and Figures S1–S4). P13 was previously identified as a taxonomic novelty by [25].

The additional strains added to the strawberry lineage in the 169-isolate tree increased its geographic range, adding China, Czechia, Japan, Germany, New Zealand and Russia to The Netherlands, Norway, Sweden, UK and USA, but largely replicated the host fidelity to *Fragaria*, suggesting strawberry production as the driver of the cosmopolitan distribution of this lineage. Based on our results, both the strawberry and apple–oak lineages are present in Czechia, Germany, Japan, The Netherlands, New Zealand, Russia, the UK and the USA. An additional *P. cactorum* subspecific lineage was identified in the 169-isolate tree—a “spikenard lineage” (Figure 3) associated with Japanese-origin isolates from *Aralia* and other plants cultivated in Japan [60].

The larger tree featured *P. cactorum* isolates from additional western US states, including Washington, Idaho, Oregon, and Utah (Figure 3). One of the three isolates from Washington State, baited from *Potentilla gracilis*, was placed within the California restoration lineage, while the other two, along with the isolate from Idaho, were placed within the apple–oak lineage on a node with two isolates from Czechia. The isolate from a Utah nursery was placed in the northern California forest lineage based solely on the cox2-spacer sequence (Figure S2), but two autapomorphic insertions in the ITS2 sequence kept it out of the lineage in the multi-locus tree. The single Oregon isolate of *P. cactorum*, from *Paeonia*, had an identical cox1 sequence to the majority of the California isolates and the ex-neotype (Figure S3), but a distinct ITS sequence kept it out of one of the named subspecific lineages. The three additional Oregon *P. pseudotsugae* isolates clustered with the strain P10218 rather than the ex-type (Figure 4).

### 3.4. Single-Locus Trees

Although the ITS provided some inter- and intraspecific distinctions, only *P. hedraiandra* represented a monophyletic clade in the ITS-only tree (Figure S1). The Oregon and California *P. pseudotsugae* isolates had distinct ITS sequences that were polyphyletic. *P. cactorum* was paraphyletic in respect to *P. aleatoria*, *P. alpina*, *P. hedraiandra*, and *P. pseudotsugae*. The cox1-only tree found *P. cactorum* and *P. pseudotsugae* to be monophyletic, but *P. pseudotsugae* was paraphyletic with respect to *P. cactorum* in the cox2+spacer-only tree (Figures S2 and S3). Only the strawberry intraspecific lineage was apparent in the cox1-only tree, with the other *P. cactorum* lineages combining or becoming paraphyletic in respect to each other. The strawberry and northern California forest lineages were not monophyletic in the cox2+spacer-only tree, forming two phylogenetic grades between the California restoration lineage and the apple–oak lineage (Figure S2). The California *P. pseudotsugae* isolates were paraphyletic with respect to the Oregon isolates in both of the single-locus mitochondrial trees (Figures S2 and S3).

### 3.5. Networks

**Figure 5:**
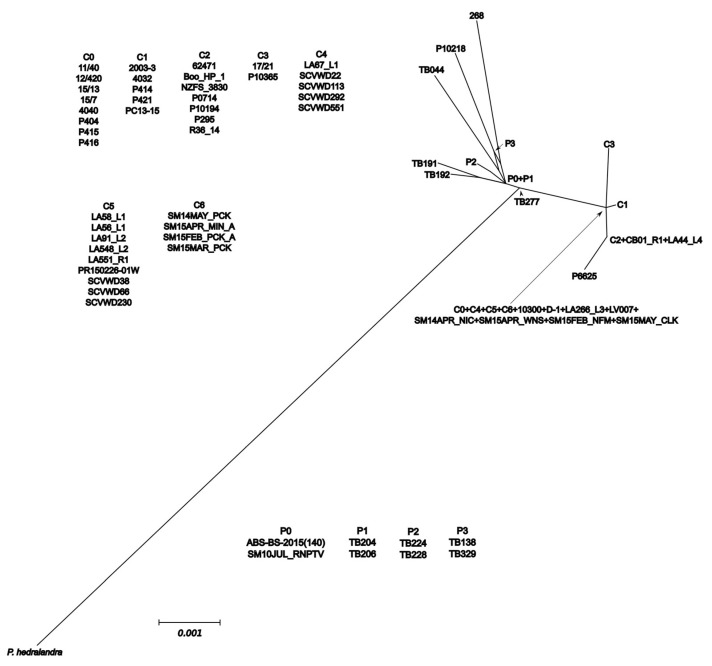
Split-decomposition network from mitochondrial cox2+cox1 loci of *Phytophthora cactorum* and *P. pseudotsugae*. Accession information listed in Table S1. Figure created with SplitsTree4 and annotated with InkScape. Scale bar is split support for node connections.

**Figure 6:**
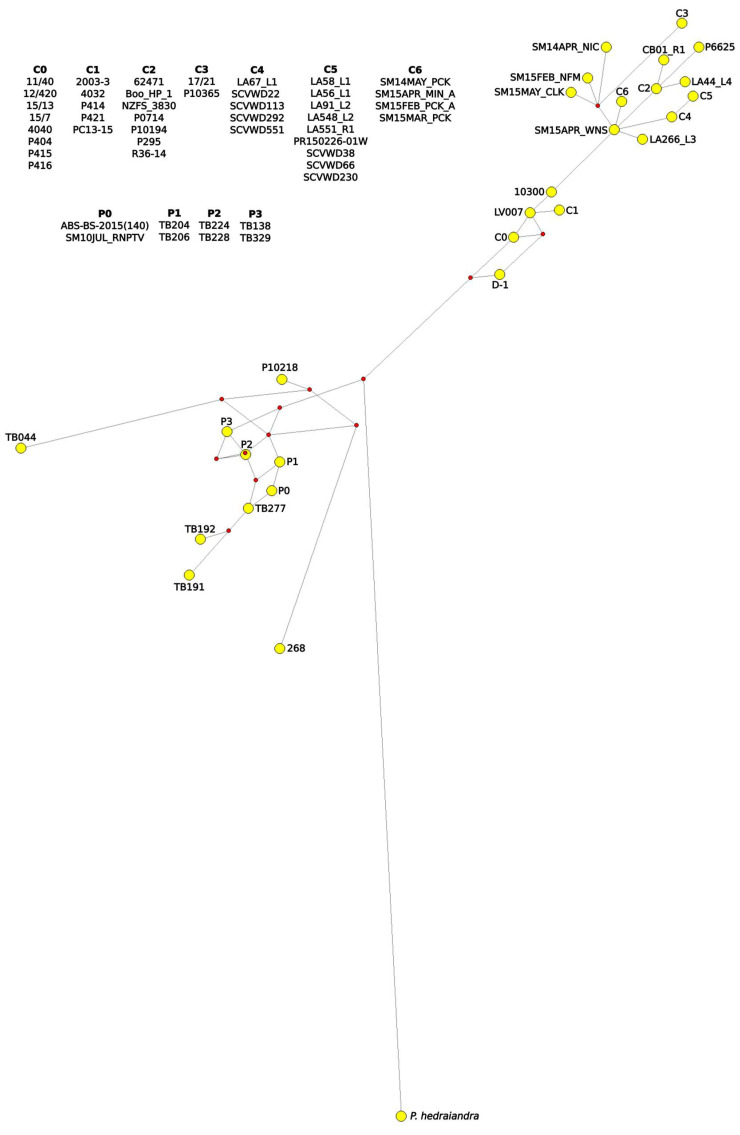
Median-joining network from mitochondrial cox2+cox1 loci of *Phytophthora cactorum* and *P. pseudotsugae*, tolerance ε = 0. Accession information listed in Table S1. Figure created with Network 10 and annotated with InkScape. Yellow circles represent isolates or groups of isolates with identical sequences, and red diamonds represent unsampled intermediates.

**Figure 7:**
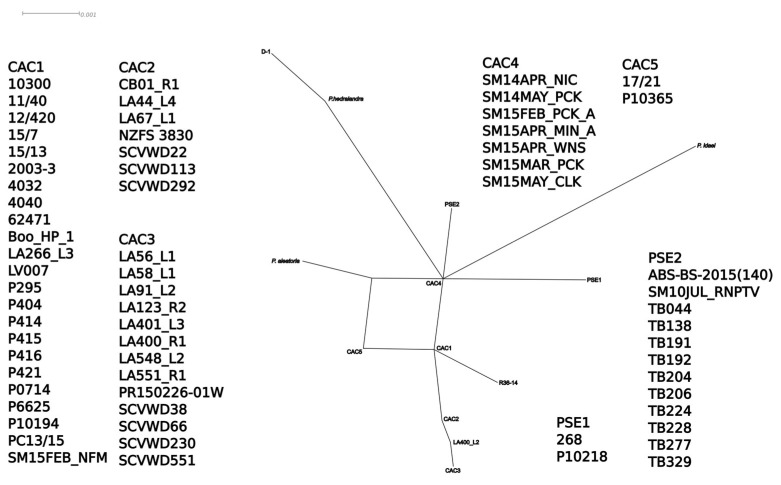
Split-decomposition network from ITS rDNA sequences of *Phytophthora cactorum*, *P. pseudotsugae* and other subclade 1a species. Accession information listed in Table S1. Figure created with SplitsTree4 and annotated with InkScape. Scale bar is split support for node connections.

**Figure 8:**
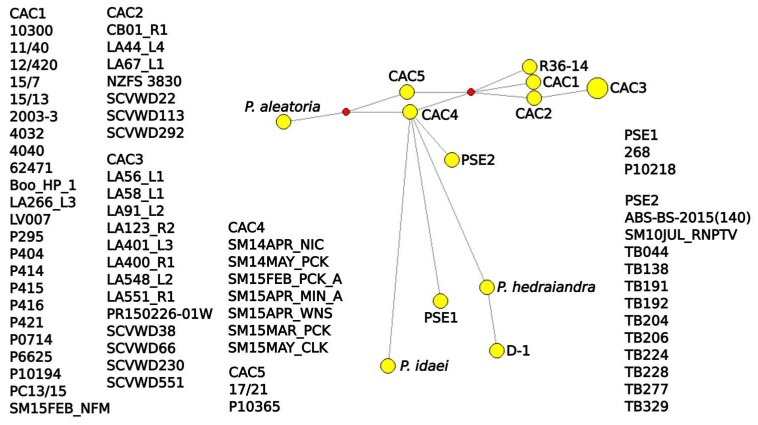
Median-joining network from ITS rDNA sequences of *Phytophthora cactorum*, *P. pseudotsugae* and other subclade 1a species. Accession information listed in Table S1. Figure created with Network 10 and annotated with InkScape. Yellow circles represent isolates or groups of isolates with identical sequences, and red diamonds represent unsampled intermediates.

The mitochondrial SDN reduced the intraspecific complexity of *P. cactorum* considerably, with only five nodes (Figure 5). A node shared by most of the strawberry crown rot isolates, the northern California stream isolates and the California restoration isolates connected *P. cactorum* to the rest of the network and had the most connections of any of the species’ nodes. One of the California soil-baited isolates, TB277, connected *P. pseudotsugae* to the rest of the network, and a node containing the single water-baited and tanoak isolates and two additional soil isolates had the most connections for that species. In the mitochondrial MJN with tolerance ε = 0, the strawberry lineage and strain D-1 formed a smaller network connecting the remainder of *P. cactorum* isolates to the rest of the network (Figure 6), but this topology was not maintained with ε increased to 10 (Figure S5). In both mitochondrial MJNs, strain SM15APR_WNS, baited from a forest stream the very northernmost of coastal California, was on a node with the most connections, an indication of ancestral status. In both the ITS SDN and MJN, the ITS haplotype corresponding to several isolates from northern California forest streams (including SM15APR_WNS) had the most connections of any node, suggesting it is ancestral within the subclade (Figure 7 and Figure 8). The topology of the ITS MJN was not altered when ε was increased to 10.

## 4. Discussion

### 4.1. Two Worldwide Lineages of P. cactorum

*Phytophthora cactorum* is widely regarded as a worldwide pathogen with a wide host range and low host specialization [2]. Our results generally confirm this characterization but strongly suggest there are at least two distinct intraspecific lineages distributed across the world, one strongly associated with strawberry production and the other with a wider range of hosts, notably apples, oak trees and woody ornamentals. Identifying a strawberry crown rot-associated lineage distinct from a lineage with a wider host range is generally congruent with previous efforts to characterize this worldwide species [20,21,24,25]. Re-creating and re-investigating these findings within a phylogenetic, barcode-based context, congruent with ongoing systematic studies, but also including recent genome-sequencing efforts allowed for greater contextualization of our California isolates, and may do the same for *P. cactorum* research worldwide.

We identified subspecific lineages in California and Japan that appear to be geographically isolated and others with much wider distributions, but it is beyond the power of this study to determine to what extent anthropogenic movement is responsible for the worldwide nature of *P. cactorum*. Nevertheless, based on our findings, it seems unlikely to observe multi-locus genotypes at these phylogenetic resolutions naturally distributed across such major geographic divisions. It is likely that the global trade of both agricultural and horticultural plants is responsible for these patterns.

Describing intraspecific diversity is difficult, and traditional phylogenetic methods are limited, even for a homothallic species such as *P. cactorum* that is predominantly asexual [26]. Although the four intraspecific lineages we identified clustered relatively consistently across our phylogenies and networks (Figure 2, Figure 3, Figure 4, Figure 5, Figure 6, Figure 7, Figure 8 and Figure S5), the topology of the relationships between the lineages was not consistent. We consider complete and near-complete matches of barcoding sequences from pathogen isolates across the world significant results, but illustrating them within bifurcating phylogenetic figures represents a compromise. The phylogenetic networks uncovered multifurcations within both species, suggesting the presence of both ancestral and derived isolates within our analysis (Figure 5, Figure 6, Figure 7, Figure 8 and Figure S5) a realistic biological possibility. This, along with a paucity of informative sites, may explain the relatively low support values for intraspecific bipartitions in the phylogenetic analyses (Figure 2, Figure 3, Figure 4 and Figures S1–S4). While a better understanding of the global population of all *Phytophthora* species is vital, molecular barcodes are not designed to facilitate analysis of populations, and insightful population analyses require the genome of each isolate to be much more thoroughly sampled.

### 4.2. Diversity of California Isolates

The intraspecific diversity observed among the local isolates suggests that both *P. cactorum* and *P. pseudotsugae* are pathogens indigenous to California (i.e., not initially introduced by humans). A northern California stream isolate, SM15APR_WNS, occupied an ancestral node in all of the intraspecific networks constructed (Figure 5, Figure 6, Figure 7, Figure 8 and Figure S5). Our evidence also suggests that the native range of *P. cactorum* also includes Japan (Figure 2, Figure 3 and Figure 4). According to our results, there are lineages within both species that are not currently present in California and, therefore, theoretically pose risks to native plants if they were to be introduced. Our evidence also suggests that at least one genotype of *P. cactorum* is being locally spread via restoration activities. In California, the native plant nurseries serving restoration needs are often separate from the nurseries serving horticulture, though the latter often sell native plants. It appears that the *P. cactorum* California restoration lineage is, at the moment, only moving locally through restoration nurseries, while the apple–oak lineage is moving locally and worldwide through horticulture. These findings highlight some of the limitations posed by regulatory efforts aimed at *Phytophthora* and other plant pathogens, which are often codified to be species-based [61]. Non-native lineages of *P. cactorum* and even *P. pseudotsugae* may pose a double-threat to California biodiversity, inasmuch as they both threaten native plants species and might potentially compete with or even displace native *Phytophthora* lineages. Non-native lineages also increase the potential for inter- and intraspecific hybridization, compounding the potential biodiversity threat [62,63,64].

### 4.3. Phytophthora ×serendipita

While *P. cactorum* is undoubtedly a wide-ranging species, our analysis suggests that a significant portion (24/145, 17%) of the isolates previously named *P. cactorum* should be more accurately determined to be *P. hedraiandra* or *P. ×serendipita*, and in a few cases could not be unambiguously determined to a described species. The nomenclatural code dictates that once a nothospecific name is applied to an interspecific hybrid, this nothospecies applies to any pairing between the two parental species [65]. The original description of *P. ×serendipita* described recent hybrids with single-nucleotide variants (SNVs) (double-peaks) in nuclear sequences and instances of both species serving as mitochondrial parents [13]. In this study, we observed strains of *P. ×serendipita* without evidence of SNVs, but with ITS sequences corresponding to one species and mitochondrial sequences corresponding to the other (Table S1, Figures S2–S4). The lack of SNVs may mean these isolates derive from older hybridization events and have had more opportunities for loss of heterozygosity and homogenization of rDNA repeats [63,64,66]. Like the original description [13], we found evidence of reciprocal pairings, although the lineage associated with *P. cactorum* mitochondrial sequences was more common, appearing to be relatively widespread across Asia and Europe and including the D-1 strain from *Panax*. It is not clear to what extent human activity is responsible for these putative hybrids. The intermediate placement of strain D-1 in the cox2+spacer only tree (Figure S2) could also be interpreted as arising from incomplete lineage sorting rather than recent hybridization [26,67,68,69]. The Eurasian *P. ×serendipita* lineage, along with several *P. cactorum* isolates from Finland (isolated from *Betula* and water) were consistently inferred to be the most early-diverging within the species (Figure 2, Figure 4 and Figures S2 and S3).

### 4.4. Sequence Barcodes and Data Sources

Genome-sequencing reads were an excellent source of data for this study, as any desired loci were available with full coverage, but required a great deal of additional effort to include these strains in an analysis. Nucleotide accessions can be downloaded and immediately analyzed, and are accessible in BLAST searches. As predicted by [31], we found the cox2+spacer locus to provide much better intraspecific resolution than the cox1, but cox1 had a much greater range of sequences available in the nucleotide collection for comparison (Figures S2 and S3, Table S1). ITS sequences alone are not sufficient to make accurate species determinations for many isolates in the species complex that is subclade 1a (Figure S1). Although there were discernable differences between groups of isolates (e.g., all California *P. pseudotsugae* strains had an identical, otherwise unique ITS sequence), in most cases these were not phylogenetically consistent enough to serve as a basis for species determinations (Figure S1). Furthermore, the existence of the *P. ×serendipita* isolates without obvious SNVs in their ITS sequences confirm that *P. cactorum* and *P. hedraiandra* cannot be accurately separated from *P. ×serendipita* with the sequence of a single locus, be it nuclear or mitochondrial [13].

### 4.5. Host Specificity of P. cactorum

While our results support the wide host range of *P. cactorum*, there is no doubt that members of the Rosaceae are particularly well-represented. This affinity is not unique within subclade 1a, as *P. idaei* possesses apparent host specialization for *Rubus* [9]. There are Rosaceae-associated isolates in all of the *P. cactorum* intraspecific lineages, except the Eurasian *P. ×serendipita* lineage (Figure 3 and Figure 4), nor any of the *P. hedraiandra* or *P. pseudotsugae* isolates included in this study. Two recent studies [24,26] found considerable differences in effector repertoires between intraspecific *P. cactorum* lineages, but this does not necessarily indicate specialization or coevolution with a particular host. There are isolates from *Fragaria* in the apple–oak lineage and from *Fagus* in the strawberry lineage, suggesting whatever host specialization may have occurred in the lineages has not considerably limited their host range. Previous studies have found strawberry isolates to be less aggressive on apple and vice versa [22,24]. Nevertheless, it is quite possible that the apparent host affinities of the apple–oak and strawberry lineages may be less about host specialization and more about the different patterns and frequencies of global plant (and soil) movement by strawberry and apple production, and in the case of the former, ornamental plant production as well. It is unfortunate that no California *Fragaria* or *Malus* isolates were able to be included in this study, and undoubtedly more isolates across the world need to be sampled. Due to the limited resolution of the barcoding loci employed in this study and the ability to directly study genes associated with pathogenicity [24,26], future work may wish to focus on accumulating additional genome-sequencing data from the worldwide population. Pathogenicity trials using several of the *P. cactorum* isolates from distinct lineages with a variety of native California host species are currently in progress.

## Figures and Tables

**Table 1:** List of projects from which the 42 California *Phytophthora* isolates were selected.

Project	Restoration Outplantings	Stream Monitoring	Chapparal and Stream Soil Baiting	Forest Soil Baiting	Direct Sampling of Seedling Roots	UCD Campus Baiting
**Range within CA**	Bay area (northern), Angeles National Forest (southern)	Central to northern	Angeles National Forest (southern)	Big Sur (central)	Big Sur (central)	Sacramento Valley (northern)
**Substrate(s)**	Rhizosphere soil and plant roots	Stream water	Rhizosphere soil	Bulk soil	*Notholithocarpus densiflorus* seedling root	Rhizosphere soil
**Bait**	Pear fruit and rhododendron leaf	Rhododendron leaf	Pear fruit and rhododendron leaf	Rhododendron leaf	N/A	Pear fruit and rhododendron leaf
**Subclade 1a species isolated**	*P. cactorum*, *P. hedraiandra*, *P. ×serendipita*	*P. cactorum*, *P. pseudotsugae*	*P. cactorum*	*P. pseudotsugae*	*P. pseudotsugae*	*P. cactorum*
**Isolates used for this study** **(Counties)**	15 (Los Angeles, 7; Santa Clara, 8)	9 (Del Norte, 7; Humboldt, 2)	5 (Los Angeles)	10 (Monterey)	1 (Monterey)	2 (Yolo)

## Data Availability

Nucleotide sequence accessions produced for this study are listed in Table S1.

## Supplementary Materials

The following are available online at https://www.mdpi.com/article/10.3390/jof8030303/s1, Figure S1: ITS-only, Figure S2: cox2+spacer-only, Figure S3: cox1-only, Figure S4: 169-isolate multi-locus tree (full), Figure S5: mitochondrial median-joining network, ε = 10, Table S1: Isolates and Sequence Accessions.
