# Supplementary material for: A Barcode-Based Phylogenetic Characterization of Phytophthora cactorum Identifies Two Cosmopolitan Lineages with Distinct Host Affinities and the First Report of Phytophthora pseudotsugae in California
Source: J Fungi (Basel). 2022 Mar 16;8(3):303. doi: 10.3390/jof8030303 (PMC8950362; doi:10.3390/jof8030303)
Supplement: Supplementary file 1 [file jof-08-00303-s001.zip › TBBPCAC_Figure_S2_cox2+spacer-only.pdf]

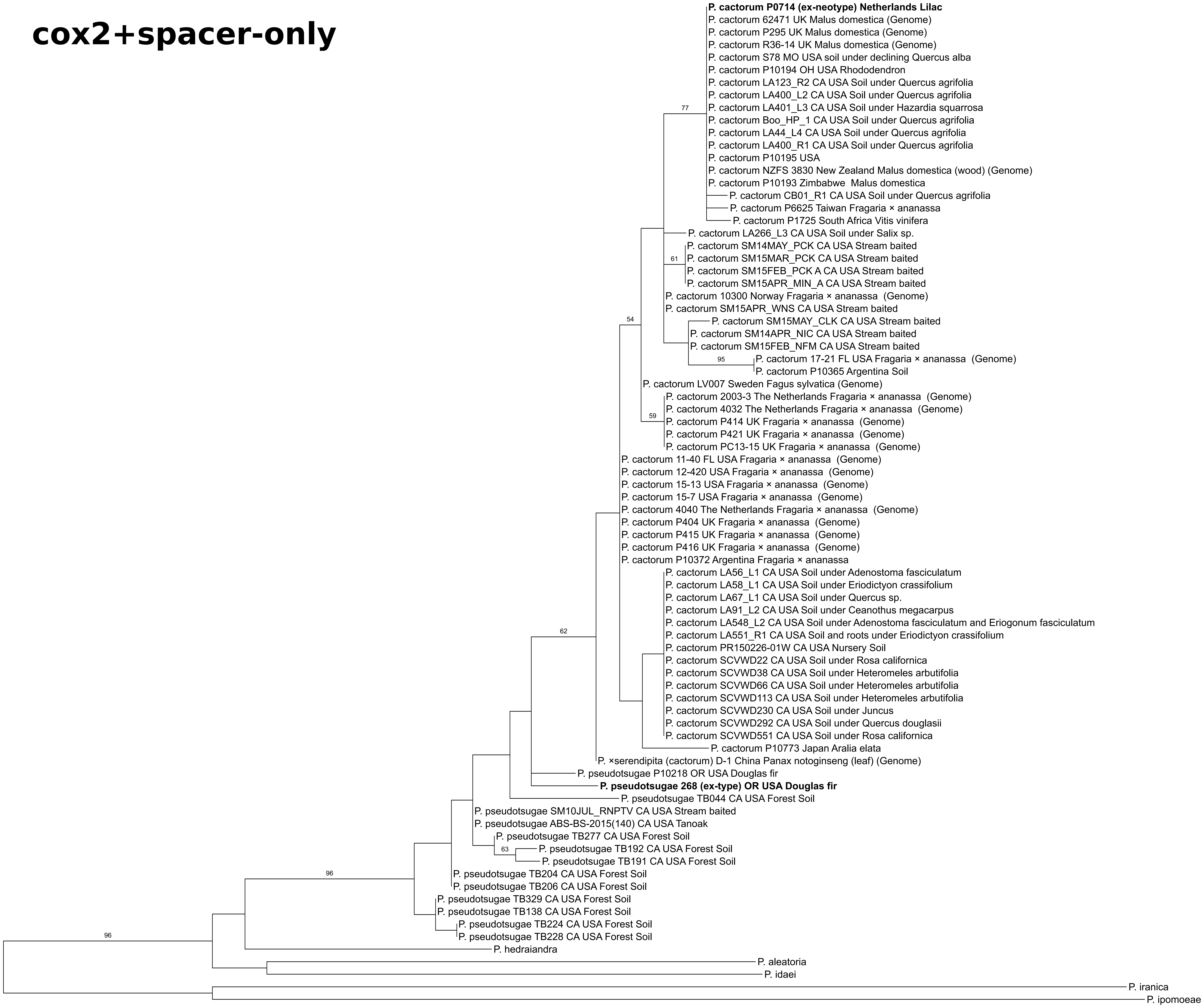

cox2+spacer-only
P. cactorum P0714 (ex-neotype) Netherlands Lilac
P. cactorum 62471 UK Malus domestica (Genome)
P. cactorum P295 UK Malus domestica (Genome)
P. cactorum R36-14 UK Malus domestica (Genome)
P. cactorum S78 MO USA soil under declining Quercus alba
P. cactorum P10194 OH USA Rhododendron
P. cactorum LA123_R2 CA USA Soil under Quercus agrifolia
P. cactorum LA400_L2 CA USA Soil under Quercus agrifolia
P. cactorum LA401_L3 CA USA Soil under Hazardia squarrosa
P. cactorum Boo_HP_1 CA USA Soil under Quercus agrifolia
P. cactorum LA44_L4 CA USA Soil under Quercus agrifolia
P. cactorum LA400_R1 CA USA Soil under Quercus agrifolia
P. cactorum P10195 USA
P. cactorum NZFS 3830 New Zealand Malus domestica (wood) (Genome)
P. cactorum P10193 Zimbabwe Malus domestica
P. cactorum CB01_R1 CA USA Soil under Quercus agrifolia
P. cactorum P6625 Taiwan Fragaria × ananassa
P. cactorum P1725 South Africa Vitis vinifera
77
P. cactorum LA266_L3 CA USA Soil under Salix sp.
P. cactorum SM14MAY_PCK CA USA Stream baited
P. cactorum SM15MAR_PCK CA USA Stream baited
P. cactorum SM15FEB_PCK A CA USA Stream baited
P. cactorum SM15APR_MIN_A CA USA Stream baited
61
P. cactorum 10300 Norway Fragaria × ananassa (Genome)
P. cactorum SM15APR_WNS CA USA Stream baited
P. cactorum SM15MAY_CLK CA USA Stream baited
P. cactorum SM14APR_NIC CA USA Stream baited
P. cactorum SM15FEB_NFM CA USA Stream baited
P. cactorum 17-21 FL USA Fragaria × ananassa (Genome)
P. cactorum P10365 Argentina Soil
95
54
P. cactorum LV007 Sweden Fagus sylvatica (Genome)
P. cactorum 2003-3 The Netherlands Fragaria × ananassa (Genome)
P. cactorum 4032 The Netherlands Fragaria × ananassa (Genome)
P. cactorum P414 UK Fragaria × ananassa (Genome)
P. cactorum P421 UK Fragaria × ananassa (Genome)
P. cactorum PC13-15 UK Fragaria × ananassa (Genome)
59
P. cactorum 11-40 FL USA Fragaria × ananassa (Genome)
P. cactorum 12-420 USA Fragaria × ananassa (Genome)
P. cactorum 15-13 USA Fragaria × ananassa (Genome)
P. cactorum 15-7 USA Fragaria × ananassa (Genome)
P. cactorum 4040 The Netherlands Fragaria × ananassa (Genome)
P. cactorum P404 UK Fragaria × ananassa (Genome)
P. cactorum P415 UK Fragaria × ananassa (Genome)
P. cactorum P416 UK Fragaria × ananassa (Genome)
P. cactorum P10372 Argentina Fragaria × ananassa
P. cactorum LA56_L1 CA USA Soil under Adenostoma fasciculatum
P. cactorum LA58_L1 CA USA Soil under Eriodictyon crassifolium
P. cactorum LA67_L1 CA USA Soil under Quercus sp.
P. cactorum LA91_L2 CA USA Soil under Ceanothus megacarpus
P. cactorum LA548_L2 CA USA Soil under Adenostoma fasciculatum and Eriogonum fasciculatum
P. cactorum LA551_R1 CA USA Soil and roots under Eriodictyon crassifolium
P. cactorum PR150226-01W CA USA Nursery Soil
P. cactorum SCVWD22 CA USA Soil under Rosa californica
P. cactorum SCVWD38 CA USA Soil under Heteromeles arbutifolia
P. cactorum SCVWD66 CA USA Soil under Heteromeles arbutifolia
P. cactorum SCVWD113 CA USA Soil under Heteromeles arbutifolia
P. cactorum SCVWD230 CA USA Soil under Juncus
P. cactorum SCVWD292 CA USA Soil under Quercus douglasii
P. cactorum SCVWD551 CA USA Soil under Rosa californica
P. cactorum P10773 Japan Aralia elata
62
P. ×serendipita (cactorum) D-1 China Panax notoginseng (leaf) (Genome)
P. pseudotsugae P10218 OR USA Douglas fir
P. pseudotsugae 268 (ex-type) OR USA Douglas fir
P. pseudotsugae TB044 CA USA Forest Soil
P. pseudotsugae SM10JUL_RNPTV CA USA Stream baited
P. pseudotsugae ABS-BS-2015(140) CA USA Tanoak
P. pseudotsugae TB277 CA USA Forest Soil
P. pseudotsugae TB192 CA USA Forest Soil
P. pseudotsugae TB191 CA USA Forest Soil
63
96
P. pseudotsugae TB204 CA USA Forest Soil
P. pseudotsugae TB206 CA USA Forest Soil
P. pseudotsugae TB329 CA USA Forest Soil
P. pseudotsugae TB138 CA USA Forest Soil
P. pseudotsugae TB224 CA USA Forest Soil
P. pseudotsugae TB228 CA USA Forest Soil
96
P. hedraiandra
P. aleatoria
P. idaei
P. iranica
P. ipomoeae
