# Supplementary material for: A Barcode-Based Phylogenetic Characterization of Phytophthora cactorum Identifies Two Cosmopolitan Lineages with Distinct Host Affinities and the First Report of Phytophthora pseudotsugae in California
Source: J Fungi (Basel). 2022 Mar 16;8(3):303. doi: 10.3390/jof8030303 (PMC8950362; doi:10.3390/jof8030303)
Supplement: Supplementary file 1 [file jof-08-00303-s001.zip › TBBPCAC_Figure_S5_mitochondrial_median-joining_network_epsilon_10.pdf]

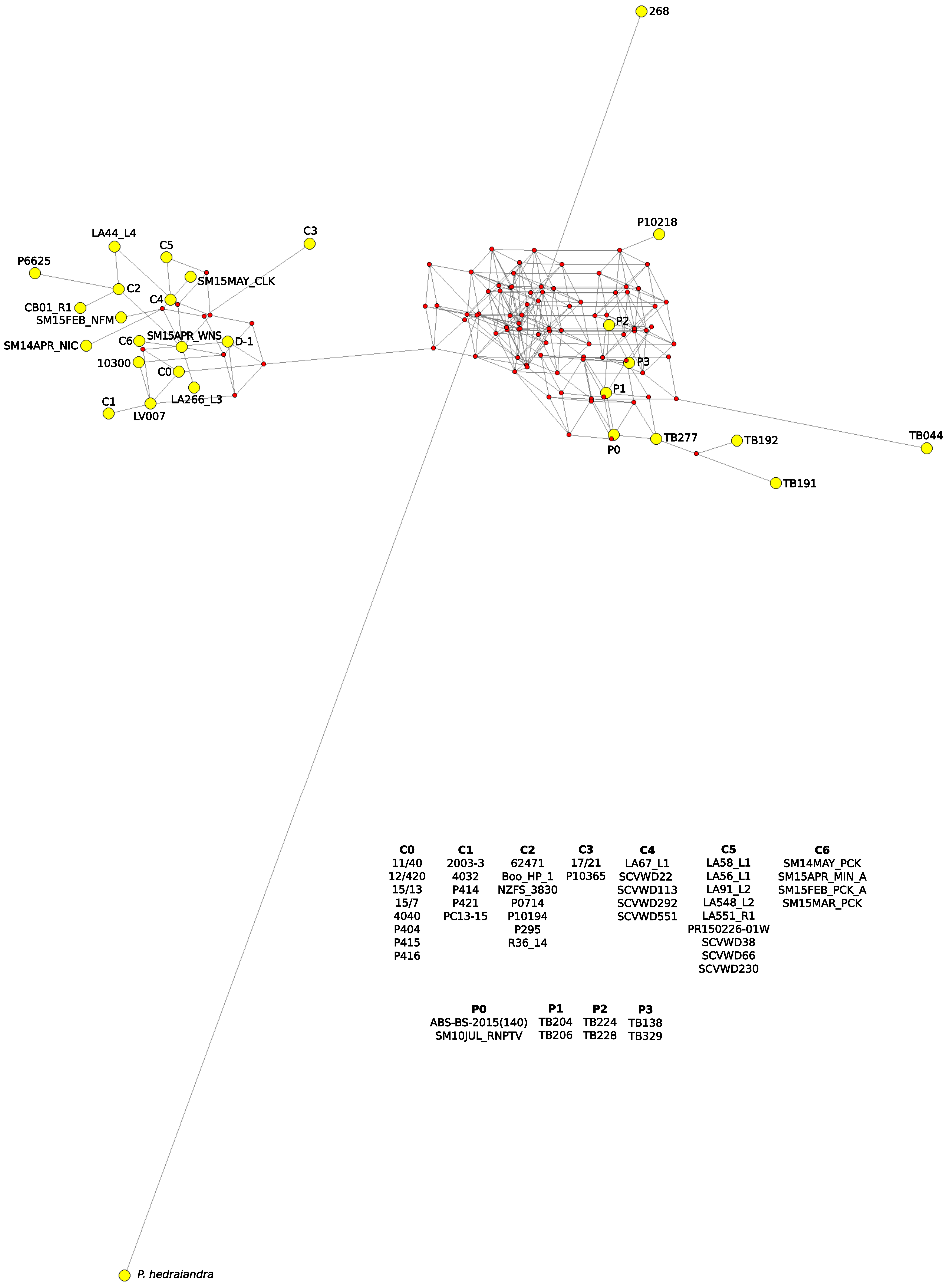
268
P10218
C3
LA44_L4
C5
P6625
SM15MAY_CLK
C2
C4
CB01_R1
SM15FEB_NFM
C6
SM15APR_WNS
D-1
SM14APR_NIC
10300
C0
LA266_L3
C1
LV007
P2
P3
P1
TB277
TB192
TB044
P0
TB191
C0
11/40
12/420
15/13
15/7
4040
P404
P415
P416
C1
2003-3
4032
P414
P421
PC13-15
C2
62471
Boo_HP_1
NZFS_3830
P0714
P10194
P295
R36_14
C3
17/21
P10365
C4
LA67_L1
SCVWD22
SCVWD113
SCVWD292
SCVWD551
C5
LA58_L1
LA56_L1
LA91_L2
LA548_L2
LA551_R1
PR150226-01W
SCVWD38
SCVWD66
SCVWD230
C6
SM14MAY_PCK
SM15APR_MIN_A
SM15FEB_PCK_A
SM15MAR_PCK
P0
ABS-BS-2015(140)
SM10JUL_RNPTV
P1
TB204
TB206
P2
TB224
TB228
P3
TB138
TB329
P. hedraiandra
