# Supplementary figures and images for: A Barcode-Based Phylogenetic Characterization of Phytophthora cactorum Identifies Two Cosmopolitan Lineages with Distinct Host Affinities and the First Report of Phytophthora pseudotsugae in California
Source: J Fungi (Basel). 2022 Mar 16;8(3):303. doi: 10.3390/jof8030303 (PMC8950362; doi:10.3390/jof8030303)

ITS-only

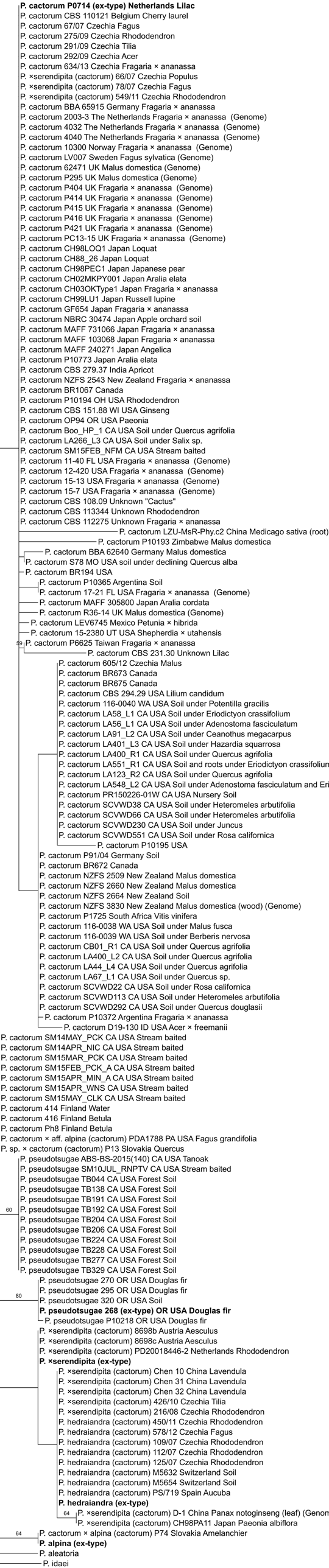

Supplement: Supplementary file 1 [file jof-08-00303-s001.zip › TBBPCAC_Figure_S1_ITS-only.pdf]

cox1-only

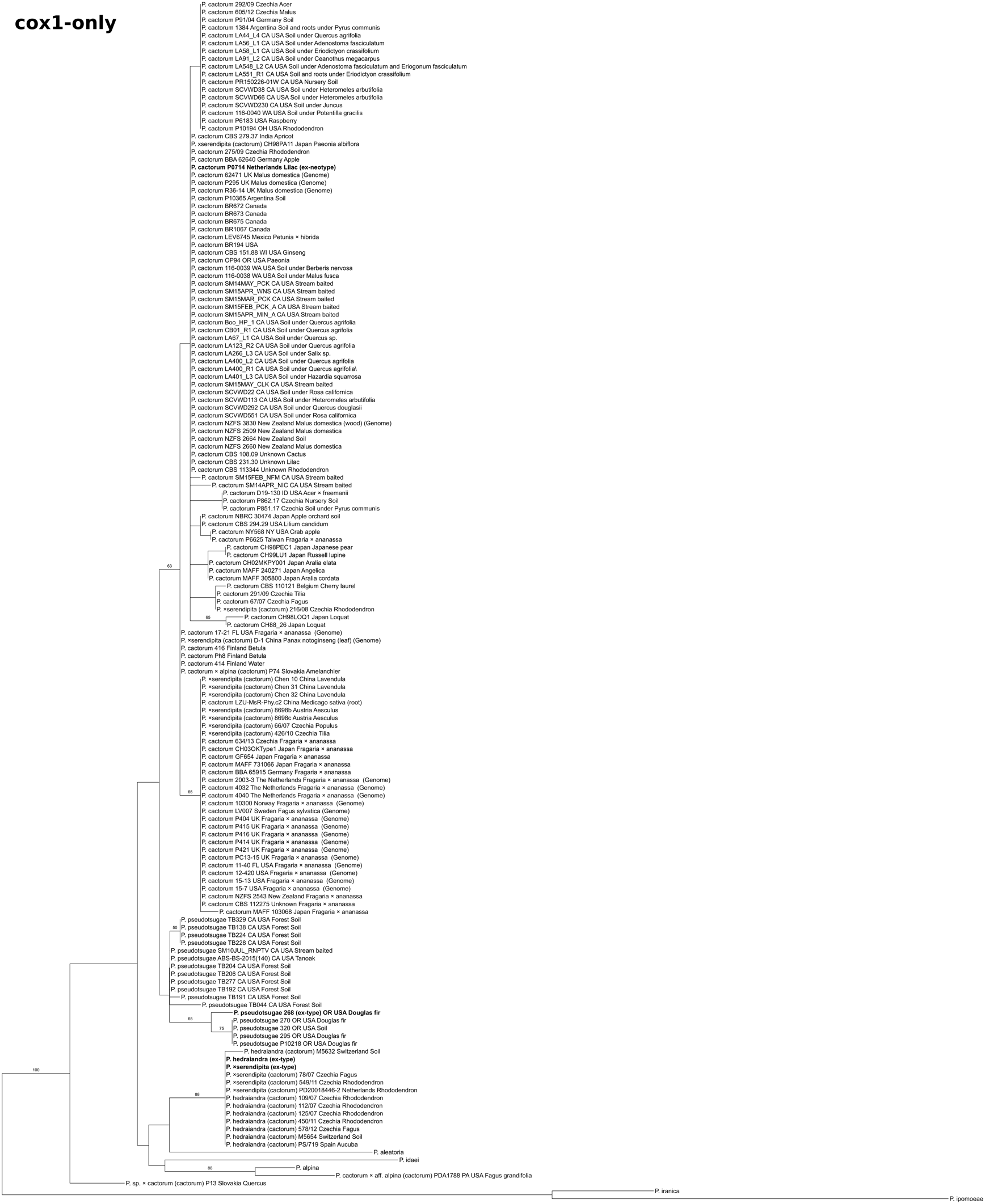

Supplement: Supplementary file 1 [file jof-08-00303-s001.zip › TBBPCAC_Figure_S3_cox1-only.pdf]

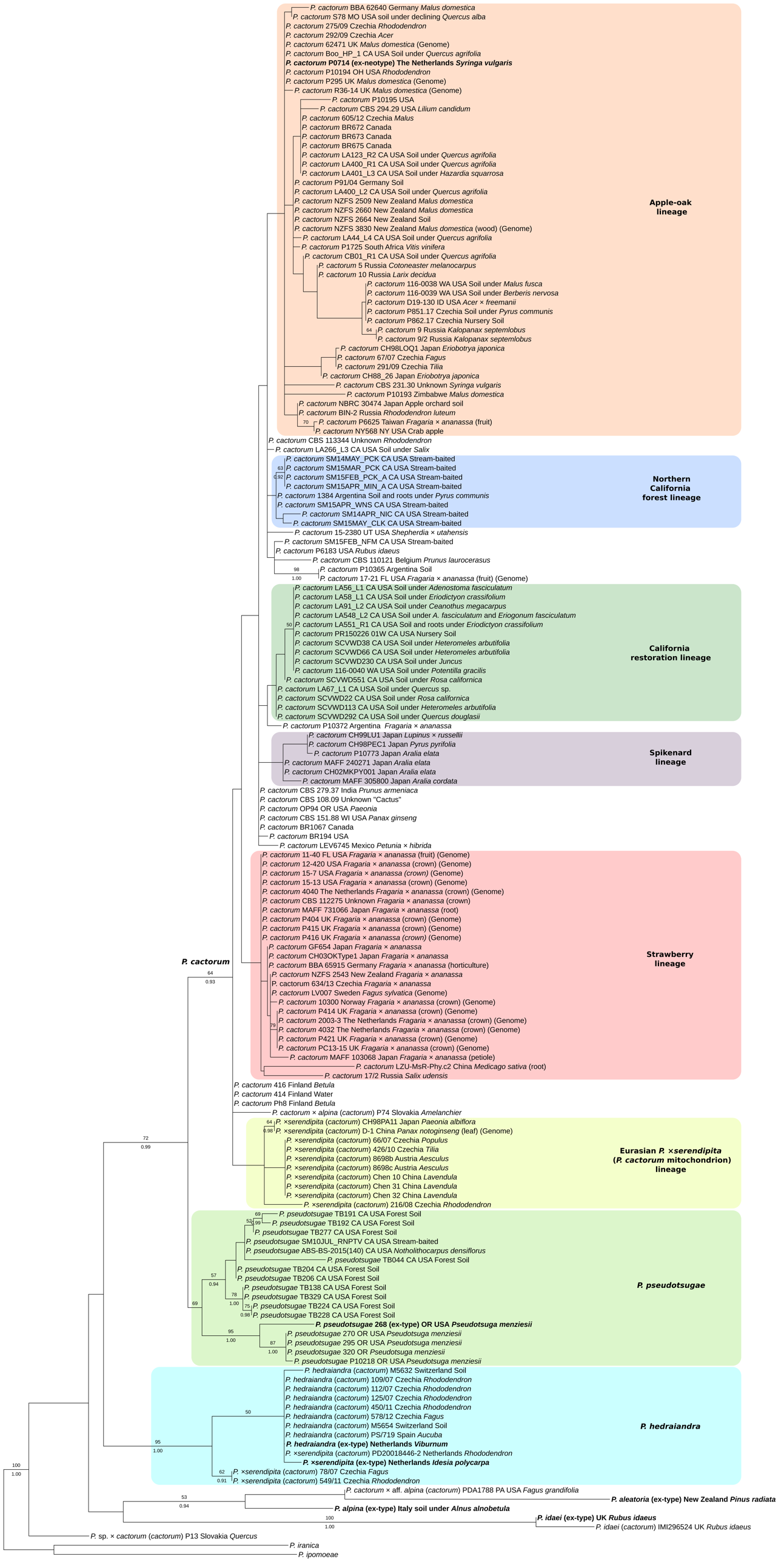

Supplement: Supplementary file 1 [file jof-08-00303-s001.zip › TBBPCAC_Figure_S4_partial_coverage_multi-locus.pdf]
